# Neighborhoods, Alcohol Outlets and Intimate Partner Violence: Addressing Research Gaps in Explanatory Mechanisms

**DOI:** 10.3390/ijerph7030799

**Published:** 2010-03-04

**Authors:** Carol B Cunradi

**Affiliations:** Prevention Research Center, Pacific Institute for Research and Evaluation, 1995 University Avenue, Suite 450, Berkeley, CA 94704, USA; E-Mail: cunradi@prev.org; Tel.: +1-510-883-5771; Fax: +1-510-644-0594

**Keywords:** alcohol outlets, neighborhoods, drinking, intimate partner violence

## Abstract

Indices of heavy drinking have consistently been linked with increased risk for intimate partner violence (IPV) among couples in the general household population. Because IPV is a ‘private’ event, most IPV research has focused on individual-level risk factors, but current social ecological theory suggests that alcohol outlets can act with neighborhood conditions to increase risks for IPV. This paper reviews the theoretical and empirical literatures relevant to identifying specific social mechanisms linking IPV to alcohol use in community settings, and discusses three social mechanisms relevant to these effects: greater numbers of alcohol outlets within a neighborhood may (1) be a sign of loosened normative constraints against violence; (2) promote problem alcohol use among at-risk couples, and; (3) provide environments where groups of persons at risk for IPV may form and mutually reinforce IPV-related attitudes, norms, and problem behaviors. Understanding these mechanisms is of critical public health importance for developing environmental strategies aimed at prevention of IPV, such as changes in zoning, community action and education, and policing.

## 1. Introduction

For couples living in socially disorganized neighborhoods, alcohol outlets can act with neighborhood conditions to increase their risks for intimate partner violence (IPV). Greater numbers of alcohol outlets within a neighborhood may be a sign of loosened normative constraints against violence; promote problem alcohol use among at-risk couples; and provide environments where groups of persons at risk for IPV may form and mutually reinforce IPV-related attitudes, norms, and problem behaviors. This paper begins with an introduction to IPV research and the role of drinking, and then reviews the theoretical and empirical literatures relevant to identifying specific social mechanisms linking IPV to alcohol use in community settings. Understanding these mechanisms is of critical public health importance for developing environmental strategies aimed at IPV prevention, such as changes in zoning, community action and education, and policing.

## 2. Methods

The PubMed database was searched (1980 to 2009) for epidemiological studies related to intimate partner violence, neighborhoods, alcohol outlets, drinking, and social disorganization. Bibliographies of certain articles provided additional papers. Articles were screened for their relevance to the specific topic of neighborhoods, alcohol outlets, and intimate partner violence on the basis of the title and abstract.

## 3. Intimate Partner Violence

### 3.1. Definition

The American Psychological Association Presidential Task Force on Violence and the Family defines domestic violence as “…the physical, sexual, and emotional maltreatment of one family member by another” [1]. While the term *domestic violence* typically encompasses all types of family violence, including elder abuse, marital rape, child sexual and physical abuse, and child psychological maltreatment, the term *intimate partner violence* (IPV) refers to those acts of aggression between adult married or cohabiting intimate partners. Aggression may occur in many ways. Psychological aggression (coercive verbal and nonverbal behaviors that are not directed at the partner’s body, such as slamming doors or smashing objects) has been found to predict physical aggression in longitudinal studies of married couples [2,3]. Physical aggression, including sexual coercion, refers to coercive acts directed at the partner’s body that may or may not cause injury. This paper focuses on physical aggression between intimate partners.

### 3.2. Prevalence & Consequences

IPV remains a significant public health problem. Based on a national probability sample of married or cohabiting couples that participated in the 1995 National Alcohol Survey, annual prevalence estimates for any partner-to-partner violence (*i.e.*, male-to-female or female-to-male) ranged from 7.8% to 21.5% [4]. A large body of research among general population samples has shown that IPV prevalence is highest among younger couples, members of racial/ethnic minorities, and couples with household indicators of lower socioeconomic status (SES), such as unemployment, lower education and income levels [5–8].

### 3.3. Male-to-Female and Female-to-Male Partner Violence

Because women are more likely than men to sustain injuries as a result of IPV [9–11], male-to-female partner violence (MFPV) has been regarded as the more urgent public health issue, and has received considerably more research attention than female-to-male partner violence (FMPV). Survey evidence from nationally representative samples, however, suggests that rates of FMPV equal or exceed MFPV rates among couples in the general household population [4,12–14], and that approximately half of IPV events are bi-directional (*i.e.*, male-to-female and female-to-male), with the remainder divided between male-to-female only and female-to-male only partner violence [15,16]. It is therefore important to address the contribution of individual- and environmental-level factors to both types of IPV in order to further public health prevention efforts.

### 3.4. IPV Typologies

Over the past decade, considerable progress has been made towards identifying different types or contexts of IPV [17–20]. For example, Johnson [18] argues that there are at least two distinct types of IPV: common couple violence and ‘patriarchal terrorism’. The former is theorized to characterize the type of situational outbursts that may occur between couples, typically in the course of conflict. Common couple violence (also known as situational couple violence) can be bi-directional, and usually involves ‘moderate’ acts (e.g., pushing, shoving, grabbing, slapping), although escalation to severe episodes (e.g., hitting with fist, kicking) is possible. Patriarchal or intimate terrorism is characterized by a pattern of more severe violence typically associated with terms such as ‘wife beating’ and ‘battered women’. This type of violence, theorized as being rooted in patriarchal ideology and tradition, is a form of terroristic control of women by their male partners. It involves the systematic use of violence, as well as other control tactics, such as threats, emotional abuse, isolation, and economic dependency [17].

### 3.5. *Common Couple Violence* vs. *Intimate Terrorism*

These distinctions are methodologically and theoretically important. Methodologically, one would expect the overwhelming majority of IPV reported by couples sampled from the general household population to consist of common couple violence [21]; cases drawn from shelter, clinical, or treatment populations are more likely to represent intimate terrorism [17]. Theoretically, the distal and proximal correlates of IPV are thought to differ based on male batterer typology [19,20]. Although its consequences are not as severe as those for intimate terrorism, situational couple violence has deleterious health consequences. For example, Johnson and Leone [17] found that women who experienced situational couple violence experienced significantly more depressive symptoms, and were significantly more likely to use antidepressants, compared to women who did not experience any couple violence. Second, common couple violence consisting of moderate acts (e.g., pushing, shoving, grabbing) can potentially progress over time to more severe levels of IPV [22]. Given that common couple or situational violence comprise most IPV events in the general population, focusing on the individual and environmental-level factors associated with common couple or situational violence has significant public health implications for IPV prevention.

## 4. Epidemiology

### 4.1. Individual Risk Factors for IPV: Drinking

Although not a “necessary or sufficient cause” of IPV, problem drinking (e.g., heavy or binge drinking; intoxication) on the part of the male often precedes or accompanies acts of IPV [23]. In addition, some research suggests that problematic drinking patterns on the part of the male and female are associated with both MFPV and FMPV among couples in the general household population [24,25]. Context of drinking and other potential moderator variables may be of critical importance for understanding why alcohol contributes to IPV for some couples under some circumstances but not others [26]. Several theoretical explanations of the alcohol-IPV relationship have been proposed. While a full discussion of these theories is beyond the scope of this article, Klostermann and Fals-Stewart [27] recently reviewed the evidence for three proposed mechanisms underlying the alcohol-IPV association: the spurious cause model in which the alcohol-IPV relationship is the result of these variables being related to other factors that influence both drinking and IPV; the indirect effects model in which alcohol use has detrimental effects on relationship quality by increasing marital discord, which in turn increases the likelihood of IPV; and the proximal effects model in which alcohol intoxication is a proximal causal agent of IPV via the psychopharmacologic effects of alcohol on cognitive processing or through alcohol-related expectancies [28].

The preponderance of evidence for the spurious cause model is weak in that the association between alcohol and IPV remains significant even when a range of psychosocial and sociodemographic variables related to both behaviors are controlled for [23]. Likewise, the indirect effects model is not well supported empirically because the alcohol-IPV association remains significant even when marital discord and similar variables are statistically accounted for [29]. Klostermann and Fals-Stewart [27] suggest that there is now considerable empirical support for the proximal effects model, including longitudinal studies that have found that the husband’s alcohol use predicted subsequent marital aggression [3,30,31]. Research conducted among male alcoholics has shown that the occurrence of IPV was significantly reduced after the men completed treatment for alcohol dependence [32]. Testa, Quigley and Leonard found that the husband’s acts of IPV that occurred when the husband was drinking involved more acts of aggression and greater severity compared to sober IPV events [33]. Despite the empirical evidence linking alcohol to IPV, it is important to note that IPV can and does occur in the absence of drinking or alcohol problems. Context of drinking and other moderator variables may be of critical importance for understanding why alcohol contributes to IPV for some couples under some circumstances but not others [26].

### 4.2. Drugs and IPV

It is important to note that illicit drug use on the part of the male and female partner is also associated with increased risk of IPV [26,34,35]. Particularly in treatment populations, high rates of IPV are found among women drug users; likewise, rates of drug use are elevated among women in domestic violence shelter populations [36]. Because drug use is typically low in general population samples, studying the effects of drug use in relation to IPV is more difficult than that of alcohol. Several mechanisms have been hypothesized. For example, women’s drug use within abusive relationships may represent attempts at self-medication [36]. Psychopharmacologic properties of particular drugs, such as cocaine, may interact with its social correlates, such as greater propensity to use violence as a means to conflict resolution, resulting in increased likelihood of partner violence on days of use [37]. Alternatively, the association of men’s and women’s drug use with IPV may represent a marker for risky lifestyle choices and personality characteristics associated with risk-taking (e.g., impulsivity) that can lead to aggression, especially in the context of couple conflict [37].

### 4.3. Psychosocial Correlates of IPV

Numerous psychosocial correlates are significantly associated with risk for IPV perpetration, victimization, or both. These include measures of impulsivity [24,38], anger expression [39,40], approval of marital aggression [12], low marital satisfaction [41,42], and family history of violence and other adverse childhood exposures [24,43,44]. Many of these factors (e.g., family history of violence, approval of marital aggression, low marital satisfaction) have been conceptualized as distal influences on the occurrence of IPV, with substance use (*i.e.*, alcohol and drugs) acting as proximal influences [35,45]. Path model analysis among married or cohabiting couples sampled from the U.S. general household population suggests that childhood experiences with violence victimization are associated with impulsivity and drinking problems later in life, all of which are associated with higher levels of IPV [38].

### 4.4. Neighborhood Influences on IPV

Violence or aggression between intimates falls under the rubric of family violence. Like child maltreatment or elder abuse, it is typically a ‘private’ event that takes place behind closed doors [46]. Because of this, most IPV research over the past thirty five years has focused on the interpersonal characteristics of one or both members of the couple. With few exceptions [47], little attention was paid to how environmental factors may influence risk for IPV. Aided by theoretical and methodological advances in multilevel research on disease risk and health behaviors [48,49], researchers have begun to examine the contribution of neighborhood factors to risk for engaging in IPV.

### 4.5. Empirical Studies

To a large extent, these studies have consisted of empirical tests of cross-sectional data demonstrating that couples residing in disadvantaged neighborhoods are at elevated IPV risk. For example, among a national sample of white, black, and Hispanic couples, Cunradi *et al.* [50] found that black couples who lived in impoverished (≥20% of households below poverty line) neighborhoods were three times as likely to report past-year male-to-female partner violence, and twice as likely to report female-to-male partner violence, than black couples who did not live in impoverished neighborhoods. White couples who lived in impoverished neighborhoods were nearly four times likelier to report female-to-male partner violence than white couples who did not live in impoverished neighborhoods. O’Campo *et al.* [51] and Cunradi *et al.* [25] found that women who lived in neighborhoods characterized by high unemployment rates were at significant risk for male-perpetrated IPV. Van Wyk *et al.* [52], in an analysis of Wave 2 of the National Survey of Families and Households, found that rates of MFPV (*i.e.*, hitting, shoving or throwing things at the partner) were lowest in the least disadvantaged neighborhoods (3.5%) and highest in the most disadvantaged neighborhoods (7.9%).

### 4.6. Perceived Neighborhood Disorder, Drinking, & Mutual IPV

In an analysis of over 18,000 married and cohabiting respondents who participated in the 2000 National Household Survey on Drug Abuse, Cunradi [13] found that the relationship between drinking level and mutual (*i.e.*, respondent report of both male-to-female and female-to-male partner violence) IPV varied by level of perceived neighborhood social disorder among women, but not men. These interactions were probed by estimating the impact of drinking level on mutual IPV conditional on neighborhood disorder being set to high and then low values, with all other variables in the model held constant [13]. The results showed that compared to women abstainers, risk for mutual IPV among women who reported recent hazardous drinking was fairly constant (Odds Ratio~6.0) across levels of neighborhood social disorder. In contrast, the magnitude of effect between drinking level and mutual IPV significantly increased under conditions of high neighborhood social disorder, but decreased to insignificant levels under conditions of low neighborhood social disorder among women in more moderate drinking categories, compared to women abstainers. In other words, women whose drinking has reached dangerous levels are at significantly elevated risk for mutual IPV regardless of their neighborhood environment; the drinking level of women at less hazardous levels puts them at significant risk only if they reside in highly disordered neighborhoods. These findings are partially explained by the dual-hazard hypothesis proposed by Fox and Benson [53], in which the accumulation and interaction of individual- and environmental-level risk factors exacerbate risk for IPV.

Among men in the study sample, however, no evidence was found for the moderating role of neighborhood social disorder. Instead, a direct effects model indicated that neighborhood social disorder was significantly associated with likelihood of men reporting past-year mutual IPV (OR = 1.61; 95% CI 1.39, 1.87). Independent effects were also seen for patterns of alcohol consumption. For example, men who were recent heavy drinkers (drank 5 or more drinks on the same occasion on each of 5 days in the past 30 days) were more than six times as likely to report mutual IPV compared to men who did not drink in the past year. Men who were recent binge drinkers (drank 5 or more drinks on the same occasion on at least one day in the past 30 days) were approximately three times as likely to report past-year mutual IPV compared to men who were past-year abstainers.

## 5. Social Disorganization Theory in Relation to IPV

Socially disorganized neighborhoods have been characterized as having three components: low collective efficacy, weak informal local friendship networks, and low participation of residents in local organizations [54]. Aggregate neighborhood factors that inhibit community social organization include concentrated disadvantage, immigrant concentration, and residential instability. Weak or nonexistent social ties among residents of such neighborhoods helps create an environment where residents are unlikely to intervene in problem behaviors, such as public drunkenness or family violence. Under these conditions, higher rates of problem behaviors will be found in neighborhoods that lack the structure or resources to either prevent or combat these problems when they arise.

### 5.1. Neighborhood Disorganization, Alcohol Outlets, & IPV

Neighborhood social disorganization may independently, and in concert with high densities of alcohol outlets, lead to IPV. IPV occurs in a social and physical context. Neighborhoods that have high levels of social disorganization have greater concentrated disadvantage, residential instability, and immigrant concentrations. These neighborhoods may also have a relatively high density of alcohol outlets. Greater levels of social disorganization and a high density of alcohol outlets may promote ‘cognitive landscapes’ that result in more aggressive behavior among area residents, both in terms of alcohol consumption and norms [55], leading to increased IPV.

### 5.2. Alcohol Outlets & IPV: Research Evidence

To date, three ecological studies have examined the contribution of alcohol outlet density to the occurrence of police-reported IPV. All three found that alcohol outlet density was significantly correlated with IPV [56–58]. Moreover, one of the studies [58] had a longitudinal design, and the findings suggest that outlet density is associated with rates of IPV over time. Because of their ecological designs, however, a major limitation of these studies was their lack of individual-level data concerning drinking, respondent characteristics, and IPV.

McKinney and colleagues recently examined the relation between alcohol outlet density and IPV, and whether binge drinking or alcohol-related problems moderated the relationship between alcohol outlet density and IPV, among a sample of 1,597 couples obtained from the general household population [59]. In adjusted multilevel analyses, they found that an increase of ten alcohol outlets per 10,000 persons was associated with a 34% increased risk of MFPV; the finding for FMPV was not significant. Moreover, they found that the relationship between alcohol outlet density and MFPV was stronger among couples reporting alcohol-related problems than those reporting no alcohol-related problems. Contrary to their expectations, on-premise alcohol outlet density was positively associated with risk of MFPV; estimates concerning off-premise outlets with MFPV and FMPV were unstable, limiting their ability to interpret the findings.

## 6. Potential Social Mechanisms

Potentially synergistic interactions of alcohol outlets with aspects of neighborhood disorganization may be related to the occurrence of IPV, but these environmentally modifiable relationships have not been systematically examined. Understanding the social mechanisms that underlie the association between neighborhood context, alcohol outlets, and the occurrence of IPV is needed in order to translate research findings into policy changes or other environmental interventions aimed at IPV prevention. The following section suggests likely mechanisms by which neighborhood conditions, in concert with alcohol outlet density, increase risk for IPV.

### 6.1. Alcohol Outlets as a Sign of Loosened Normative Constraints against Violence

Greater alcohol outlet density, especially in disorganized neighborhoods, may contribute to increased IPV risk through a number of pathways. For example, Bennett *et al.* [60] suggest that alcohol outlets, particularly off-premise packaged goods liquor stores, are often surrounded by signs of physical disorder, such as empty or broken bottles, loiterers, and publicly intoxicated patrons. Together with other deleterious neighborhood conditions, the presence of alcohol outlets signals to residents that the mechanisms of informal social control are not working [60–62]. Under such conditions, residents may be less likely to become involved if they witness or hear a couple involved in IPV, either through personal intervention, calling the police, or through any sort of public acknowledgement of the IPV behavior [52]. Lack of informal social control may also lead residents of disorganized neighborhoods to be less concerned about social consequences of engaging in IPV (e.g., neighbor or police intervention), and therefore less constrained in their behavior towards their spouse or partner. Furthermore, residents in these neighborhoods may be unwilling to interfere in domestic conflicts among their neighbors due to community nonintervention norms concerning “family” or “private” disputes [63].

### 6.2. Alcohol Outlets Promote Problem Alcohol Use among At-Risk Couples

Especially for couples in socially disorganized neighborhoods, it is quite plausible that greater alcohol availability provided by bars and off-premise packaged goods stores will result in heavier drinking on the part of one or both members of the couple, and thereafter increased IPV risk. Alcohol availability theory [64] proposes that as the physical availability of alcohol increases, so too will actual alcohol use at the individual level. Thus, a relatively high concentration of bars and/or liquor stores in a particular area may increase the risk of violence such as IPV. An individual whose barriers to aggression are lowered when drinking may not have the same opportunity in a low density area compared to a high density area and thus IPV may be less. The disproportionate distribution of off-premise liquor stores in low-income African American communities may exacerbate this potential by providing a ready source of alcohol that is marketed for immediate consumption in chilled, large bottles [65]. Couples residing in neighborhoods that have greater outlet density may adopt patterns of venue use associated with heavier drinking that results in higher levels of IPV, and these patterns of venue use may be greater in neighborhoods characterized by higher levels of social disorganization.

### 6.3. Alcohol Outlets Provide Environments where High-Risk Groups Form

Greater numbers of alcohol outlets within a neighborhood may provide environments where groups of persons at risk for IPV may form and mutually reinforce IPV-related attitudes, norms, and problem behaviors. A number of scenarios are possible. For example, men who drink in bars that have physical or social characteristics that makes violence more likely [66] may return home to their spouse/partner in a disinhibited, aggressive state in which conflict can rapidly escalate to IPV. Another possibility is that the opportunity afforded to drink by the presence of off-premise outlets increases the chances that some men will purchase alcohol, consume it in the company of other intoxicated men in a public setting (e.g., street corner, park), and thereafter return home in a disinhibited, aggressive state that likewise makes IPV probable in the context of spouse/partner conflict. In addition, bars and off-premise liquor stores may help promote and/or strengthen aggressive norms. Barriers to aggression may be lowered not only by actual alcohol use but also by drinking in a setting that poorly regulates or encourages aggression. The niche theory and assortative drinking hypotheses posit that alcohol sellers ‘niche market’ to select social strata; drinkers return to outlets frequented by people like themselves; and consequent social stratification of drinkers across contexts will result in greater levels of problems in some outlets [67]. Social disorganization theory [54] suggests that higher rates of ‘deviant’ behavior, such as public intoxication and IPV, will be found in disorganized neighborhoods that lack a structure to help maintain social controls over these problem outcomes. Through these mutually reinforcing mechanisms, the presence of alcohol outlets in socially disorganized neighborhoods may compound both the effects of social disorganization and patterns of venue use and drinking. Furthermore, ambiguous or even supportive norms concerning the use of force or violence to resolve disputes may be sanctioned in socially disadvantaged neighborhoods [55,68,69].

## 7. Conclusions

### 7.1. Future Directions

The current state of IPV research suggests that couples living in socially disorganized neighborhoods are at increased risk for IPV compared to couples that do not live in socially disorganized neighborhoods, net of other individual- and couple-level characteristics. Increased alcohol outlet density appears to be associated with risk for MFPV, and this association varies depending on the presence of alcohol-related problems among the couple. Future IPV studies need to identify the mechanisms as suggested in this paper that underlie these associations. Ideally, such studies will take into account reports about IPV and drinking from both members of the couple, and will be able to assess exposure to neighborhood characteristics and alcohol outlet density over time in order to establish temporality. Collecting dyadic data has several advantages over data obtained from one partner per couple. First, dyadic data allows for the drinking behaviors and other characteristics of both partners to be modeled. Second, IPV prevalence estimates based on dyadic reports helps reduce bias associated with estimates based on reports from one partner per couple [38,70]. Multilevel studies of IPV need to account for spatial autocorrelations (measurement error related to the spatial proximity of sampled units one to another that can bias statistical estimates of effects) using techniques to control for potential Type 1 error (as in positive spatial autocorrelation) [71] or Type II error (as in negative spatial autocorrelation) [72]. Attention to geographic unit is also important. Key conceptual and methodological challenges include defining the geographic area (e.g., ‘neighborhood’) whose characteristics may be relevant to the outcome or processes under study, and operationalizing areas in a way that allows linkage of administrative data and individual-level data [73]. Some researchers have suggested that sub-divisions of cities, such as Census tracts, may be the most appropriate geographic unit to investigate the relationship between alcohol availability and violence [74]. To date, significant associations between alcohol outlet density and IPV have been identified at the postcode or zip code level [58,59]. Additional research is needed to determine the geographic unit of analysis that is conceptually and methodologically best suited to testing the hypothesized associations between neighborhood characteristics, alcohol outlets, and IPV. Finally, although McKinney and colleagues [59] did not find a significant association between alcohol outlet density and FMPV, this issue warrants further investigation. Similarly, level of IPV severity in relation to alcohol outlets and neighborhood conditions needs to be explored. Future research will also need to test whether there are gender differences in the impact of neighborhood social disorganization and alcohol outlet density on IPV, as suggested by the findings of Cunradi [13].

### 7.2. Environmental IPV Prevention Strategies are Needed

Despite progress that has been made over the past decades in understanding the factors that put couples at risk for engaging in IPV, little progress has occurred in the area of prevention. Since marital aggression, by its definition, takes place between intimates apart from public surveillance, most research has focused on the interpersonal characteristics that put couples at risk for engaging in IPV. But just as environmental strategies aimed at reducing alcohol-related problems are most effective at a population level (e.g., raising the minimum drinking age to age 21 from age 18; lowering legal blood alcohol concentration (BAC) limits; enforcement of underage sales to minors laws) [64], environmental strategies may be most effective on a population level for reducing and preventing IPV. In this regard, a recent review by Popova *et al.* concluded that restricting availability of alcohol (*i.e.*, alcohol outlet density; hours and days of sale) is an effective measure to prevent alcohol-attributable harm [75]. Understanding the environmental context in which drinking and IPV occurs can lead to the design of prevention and intervention efforts that address the confluence of individual and community factors that may put couples at elevated risk for IPV. Such an approach to prevention and intervention may therefore be a promising strategy for reducing IPV occurrence.
